# Effect of *Ageratum fastigiatum* on Viability, Migration and Proliferation of Breast Cancer Cells in 2D and 3D Culture Models

**DOI:** 10.1007/s12010-026-05589-x

**Published:** 2026-02-07

**Authors:** João Paulo de Jesus Vieira, Ilva de Fátima Souza, Marcelo Bráulio Pedras, Elton Diêgo Bonifácio, Michaelle Geralda dos Santos, Bethânia Alves de Avelar Freitas, Libardo Andrés González Torres

**Affiliations:** 1 School of Medicine, Federal University of Jequitinhonha and Mucuri Valleys, Diamantina, Minas Gerais Brazil; 2 Science and Technology Institute, Federal University of Jequitinhonha and Mucuri Valleys, Diamantina, Minas Gerais Brazil

**Keywords:** *Ageratum fastigiatum*, Cytotoxicity, Cell migration, Microfluidic device

## Abstract

Triple-negative breast cancer (TNBC) is an aggressive subtype with limited therapeutic options, underscoring the need for novel antitumor agents and more physiologically relevant in vitro models. Although species of the *Ageratum* genus exhibit biological activity, the antitumor potential of *Ageratum fastigiatum* and its evaluation in three-dimensional (3D) microenvironment have not been previously investigated. This study evaluated the cytotoxic, antiproliferative, and antimigratory effects of the ethanolic extract of *Ageratum. fastigiatum* on TNBC cells MDA-MB-231 using conventional two-dimensional (2D) cultures and a 3D tumor-on-a-chip model. In 2D cultures, cell viability assessed by the MTT assay revealed an IC_50_ of 308 µg/mL, and approximately 80% reduction in viability at a concentration of 500 µg/mL. The extract significantly inhibited cell migration in scratch assays and reduced colony formation in clonogenic assays. Cell cycle analysis showed an increased sub-G0/G1 population at the IC_50_ concentration, indicating apoptosis-associated cell cycle disruption. In the 3D microfluidic model, tumor cells embedded in a collagen hydrogel exhibited reduced sensitivity to the extract compared to 2D cultures, with viable cell areas of approximately 15%, compared to 20% under control group , highlighting the influence of the extracellular matrix on treatment response. Additionally, the extract inhibited cell migration by approximately 59% in the 3D model at the IC_25_ concentration (289 µg/mL). In conclusion, the ethanolic extract of *A. fastigiatum* demonstrated antitumor activity against TNBC cells, and the incorporation of 3D microfluidic models highlights the importance of tumor microenvironment based platforms for a more physiologically relevant evaluation of therapeutic candidates.

**Supplementary Information:**

The online version contains supplementary material available at 10.1007/s12010-026-05589-x.

## Introduction

Cancer is one of the leading causes of global mortality, representing a significant challenge to healthcare systems worldwide [1]. In 2020, cancer was responsible for approximately 10 million deaths and 19.3 million new cases, highlighting the growing impact of the disease on the global population [2]. Among the various types of cancer, breast cancer stands out as the leading cause of cancer-related death among women in developed countries, reflecting both its high incidence and severity [3]. Globally, it is the second leading cause of cancer-related mortality among women, emphasizing the importance of intensifying research for the development of more effective treatments and advanced therapeutic strategies [4].

Triple-negative breast cancer (TNBC) is one of the most aggressive subtypes of the disease, characterized by the absence of estrogen, progesterone, and human epidermal growth factor receptor 2 (HER2) on the surface of tumor cells [5–7]. Mutations in p53 protein and the lack of these receptors make TNBC more difficult to treat, as therapies targeting these receptors, such as hormone therapy and HER2 targeted treatments, are ineffective [8]. Due to this resistance, combination treatments such as chemotherapy, radiotherapy, and immunotherapy are being used [9]. These treatments cause numerous side effects, impacting the patient’s quality of life. In response to these challenges, many researchers are investigating natural compounds with anticancer properties to develop new therapeutic alternatives that may be more effective [2].

Currently, some widely used anticancer drugs are plant-derived, including taxol, vinblastine, vincristine, podophyllotoxin, and camptothecin [10]. There are also plants that have not been sufficiently explored and that could help in the treatment of cancer. Examples of this are many species of the *Ageratum* genus, belonging to the *Asteraceae* family, which are recognized for their well-documented anti-inflammatory properties [11–13]. In the Brazilian cerrado, *Ageratum conyzoides* is one of the most studied due to its unique chemical and biological characteristics [14]. In vitro andin vivo studies have demonstrated the anticancer potential of *A. conyzoides* [15, 16]. However, to date, no studies have investigated the antitumor activity of *Ageratum fastigiatum* (Gardn.) R. M. King & H. Rob., revealing a scientific gap and the need to explore the therapeutic potential of this species.

The aim of this study was to investigate the antitumor potential of *Ageratum fastigiatum* in triple-negative breast cancer cells, using two-dimensional (2D) and three-dimensional (3D) culture models that more faithfully replicate the tumor microenvironment. To this end, cytotoxicity, cell migration, and colony formation assays were performed to assess the biological effects of the plant on cancer cells.

## Methods

### Sample Collection and Preparation of Plant Extract

**Fig. 1:**
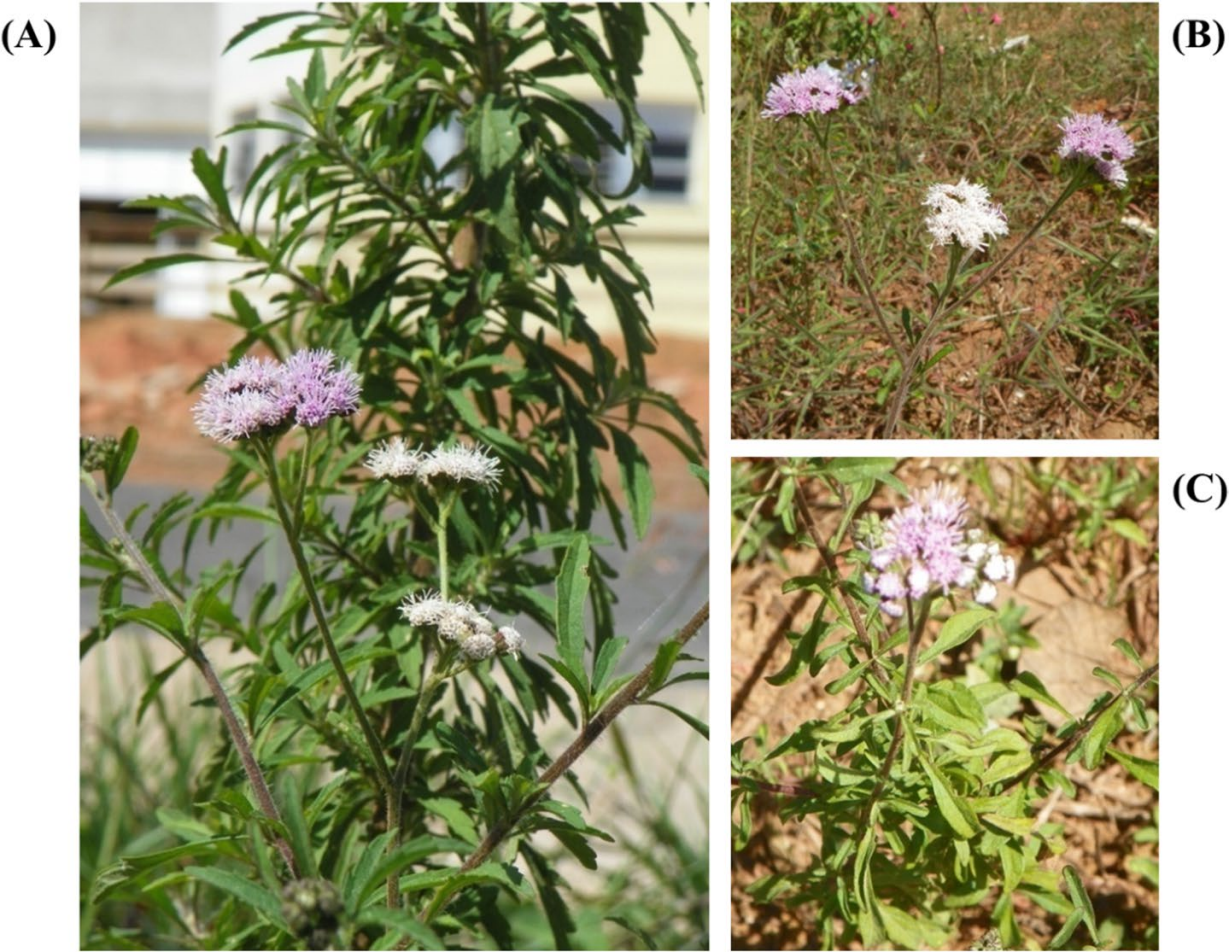
Image illustrating different parts of the plant *Ageratum fastigiatum*. (**A**) General view of the plant structure, highlighting its size and formation, along with details of the leaves, emphasizing their shape, margins, and arrangement along the stem. (**B**-**C**) Close-up view of the inflorescence, showing the arrangement of flowers and characteristics of the floral cluster

The aerial parts of the plant *Ageratum fastigiatum* (Gardn.) R. M. King & H. Rob., including the branches with leaves and inflorescences, were collected at the Juscelino Kubitschek Campus - UFVJM (S18º12.125′ W 43º34.367′, altitude 1392 m), located in the municipality of Diamantina, Minas Gerais (Fig. 1). A voucher specimen was deposited in the DIAM Herbarium of the Federal University of Jequitinhonha and Mucuri Valleys (UFVJM), under registration number 1300. The aerial parts of the plant were dried in a circulating air oven at 40 °C. The plant material was ground in a knife mill (Marconi^®^) and macerated in a 1:5 (m/v) ratio with ethanol (96% v/v). The extract was filtered through cloth and cotton filters and concentrated using a rotary evaporator (Fisatom^®^) at a temperature of 40–42 °C under reduced pressure. It was then stored in a glass container protected by aluminum foil and kept away from direct sunlight. For the experiments, a stock solution of the ethanolic extract of the aerial parts of the plant was prepared at a concentration of 200 mg/mL, dissolved in dimethyl sulfoxide (DMSO, Sigma-Aldrich^®^).

### Cell Culture and Maintenance

The human triple-negative breast cancer adenocarcinoma cell line (MDA-MB-231) was maintained in high glucose dulbecco’s modified eagle medium (DMEM; Gibco^®^), supplemented with 10% fetal bovine serum (FBS; Sigma-Aldrich^®^), 1% L-glutamine (Gibco^®^), and 1% antibiotic (100 U/mL penicillin and 100 µg/mL streptomycin). The cells were cultured in tissue culture flasks (Kasvi^®^) at 37 °C in a humidified atmosphere containing 5% CO_2_. Subculture was performed when the cells reached high confluence, they were washed twice with phosphate-buffered saline (PBS), detached with trypsin-EDTA, homogenized with culture medium, and incubated at 37 °C with 5% CO_2_.

### Cell Viability Assay in 2D Model

Cell viability was evaluated using the colorimetric 3-[4,5-dimethylthiazol-2-yl]−2,5-diphenyl tetrazolium bromide (MTT; Sigma-Aldrich^®^) assay. MDA-MB-231 cells were seeded at an initial density of $\:2.5\times\:{10}^{4}$ cells/well in 96-well plates and incubated with treatments for 72 h at 37 °C in a 5% CO_2_ atmosphere.

The standard drug, doxorubicin (DOX; Fauldoxo^®^), was used as a positive control for cell death in tumor cells, tested at the previously determined IC_50_ concentration of 7.35 µg/mL. The dimethyl sulfoxide (DMSO; Sigma-Aldrich^®^) was used as a negative control at the maximum concentration of 0.250% in the well (see supplementary material).

The cytotoxicity of the ethanolic extract of *Ageratum fastigiatum* aerial parts was tested at various concentrations (3.90, 7.81, 15.62, 31.25, 62.5, 125, 250 and 500 µg/mL). After the incubation period, treatments were removed and replaced with 20 µL of MTT (5 mg/mL) for 4 h at 37 °C in a 5% CO_2_ atmosphere. Subsequently, the formazan crystals were dissolved in 200 µL of DMSO, and absorbance was measured using a microplate reader at 570 nm. Experiments were performed in triplicate and on three different days to ensure experimental reproducibility. The percentages of cell viability and the theoretical inhibitory concentrations (IC_25_ and IC_50_) were determined using the software GraphPad Prism version 8.

### Scratch Assay To Assess Cell Migration in a 2D Model

The migration rate of MDA-MB-231 cells was evaluated using the scratch assay method. MDA-MB-231 cells were seeded in 6-well plates at a density of $\:1.0\times\:{10}^{6}$ cells/well and cultured in growth medium until they formed a confluent monolayer. Scratches were created using a P-200 micropipette tip, and cellular debris was gently removed with PBS. The cell monolayers were incubated with the plant extract (at IC_25_ and IC_50_) and doxorubicin (at IC_50_). Images were taken using optical microscopy at three different regions of the scratch at 0 h, 24 h, 48 h, and 72 h (Zeiss LSM 700). The experiment was performed in duplicate, and images captured with a 10x objective were analyzed using ImageJ^®^ software with the Wound Healing Size Tool plugin to quantify the scratch area. The results were expressed as the mean reduction in the area at different time points and graphically represented using GraphPad Prism. A three-way ANOVA with Tukey’s post-hoc test was applied for comparison between groups. To determine the scratch closure rate, the percentage reduction (%) was calculated as:

$$Wound\;closure\;(\%)\;=\;\frac{W_i-W_f}{W_i}\times\:100\%$$

W_i_: initial wound area, W_f_: final wound area.

### Clonogenic Assay in 2D Model

In a 6-well plate, MDA-MB-231 cells were seeded 500 cells/well and incubated for 24 h. Subsequently, the cells were treated with the plant extract (IC_25_ and IC_50_) and doxorubicin (IC_50_), and incubated with the treatments for 72 h. After this period, the culture medium was removed, and the wells were washed twice with PBS before adding fresh culture medium and incubating for an additional 12 days. The colonies formed were fixed with 3.7% formaldehyde for 15 min and stained with crystal violet (0.5% w/v) for 30 min at room temperature. The wells were washed under running water to remove excess dye. The experiment was performed in triplicate, and groups with 50 or more cells were considered as colonies.

The percentage of the area occupied by colonies was quantified using ImageJ^®^ software. For this, the images of the wells were converted to grayscale (8-bit), and then a selection was made using the circle tool for each well, adding them to the ROIs (Regions of Interest). Subsequently, the non-interest external areas were excluded, and a binary mask was generated using the adaptive local thresholding method with the Bernsen method. Finally, the percentage of the area occupied by colonies in each ROI was calculated individually.

### Cell Cycle Analysis by Flow Cytometry

To determine the DNA content at different phases of the cell cycle, cells were seeded in 6-well plates at a density of 1.0 × 10^5^ cells/mL and incubated for 24 h at 37 °C in a humidified atmosphere containing 5% CO₂ to allow adhesion. Subsequently, cells were treated with *A. fastigiatum* extract at IC_25_ and IC_50_ concentrations for 72 h. Following treatment, cells were detached with trypsin-EDTA, washed with 1X PBS, and centrifuged at 300 × g for 7 min at 18 °C. The cell pellets were then incubated for 30 min at 4 °C in the dark with 1 mL of fluorescent hypotonic solution containing 20 µg/mL propidium iodide (Sigma, St. Louis, MO, USA), 0.1% Triton X-100, and 0.1% sodium citrate.

Samples were analyzed using a FACSCANTO II flow cytometer (Becton Dickinson, San Jose, CA, USA), with acquisition of 20.000 events per sample, controlled by FACSDiva software (Becton Dickinson). Cell cycle distribution was determined using FlowJo software version 10.0.7.

### 3D Cell Cultures

MDA-MB-231 cells were trypsinized and resuspended in culture medium to prepare a stock solution with a final concentration of $\:1.0\times\:{10}^{6}$ cells/mL in hydrogel. For collagen hydrogel preparation, reagents were kept on ice, and the following components were used: 19 µL of dulbecco’s phosphate buffered saline (DPBS) 10X with phenol red; 100 µL of type I collagen (5.0 mg/mL, Cultrex^®^ 1706431); 66 µL of sterile Milli-Q water; 10 µL of cells; and 5 µL of NaOH 0.5 N. Type I collagen was chosen to mimic the extracellular matrix, and a final concentration of 2.5 mg/mL was used for the experiment. Using a P-10 tip, 10 µL of the hydrogel mixture was injected into the central region of a microfluidic device (AimBiotech^®^; IdenTx 3 Chip), which was incubated for 30 min at 37 °C with 5% CO_2_ to allow collagen polymerization. After this incubation period, culture medium was perfused through the side channels to allow the diffusion of oxygen and nutrients required for cell survival. The device was incubated for 4 days before applying the treatments with daily medium renewal.

After 72 h of culture, the culture medium from the side channels was removed, and the cells were treated with 500 µg/mL of *A. fastigiatum* extract (approximately IC_80_) and 7.32 µg/mL of doxorubicin (IC_50_), along with a control group without treatment. The IC_80_ concentration of the plant extract was selected for the 3D assays to obtained a more significant effect in the 3D culture. Matrix–associated barriers often difficult the diffusion of the substances and reduce the observed effect of the drug. Thus, a higher concentration could better demonstrate the effect of the studied plant extract. Treatments were renewed daily and maintained for 72 h, after which cells were stained with Calcein-AM and Propidium Iodide (PI) from a live/dead viability kit (Sigma-Aldrich^®^; Ref: CBA415). Fluorescence images were captured using a confocal microscope (Zeiss LSM 700).

The fluorescence intensity and the total area occupied by live and dead cells in the microdevice were quantified using ImageJ^®^ software. Fluorescence was measured from an image captured with the pinhole opened, approximately in the central region of the microdevice, and the results were represented in profile graphs. Fluorescence profiles were generated from the overall image fluorescence.

To quantify the total area of the cells, a z-stack with 10 slices was generated, and the total area was determined by summing the areas occupied by cells in each slice. The rolling ball method was applied to remove the image background, considering the approximate size of a cell. Then, automatic thresholding was used for segmentation generating a binary mask on the original image. This approach allowed the quantification of both fluorescence intensity and the total area occupied by cells. Results were normalized between 0 and 1, and the comparison of the total area occupied by live and dead cells was performed using the Student’s t-test.

### Cell Migration Assay in 3D Culture Model

A collagen hydrogel solution with pH 7.4 was prepared using 20 µL of DPBS 10X with phenol red, 100 µL of type I collagen (5.0 mg/mL, Cultrex^®^ 1706431), 73 µL of sterile Milli-Q water, and 7 µL of NaOH 0.5 N. Then, 10 µL of this solution was injected into the central microchamber, and the device was incubated for 30 min at 37 °C in an atmosphere with 5% CO₂. Meanwhile, MDA-MB-231 cells were resuspended and adjusted to a concentration of $\:3.0\times\:{10}^{6}$cells/mL.

After hydrogel incubation, 120 µL of unsupplemented culture medium was added to one of the side channels, along with 10 µL of cells in each of the two entries of the channel, totaling 140 µL of medium with cells. In the opposite side channel, 100 µL of unsupplemented culture medium was added, creating a pressure difference to direct the cells to the collagen hydrogel surface. The microfluidic device was incubated again for 3 h.

The effect of *A. fastigiatum* was evaluated at a concentration of 289 µg/mL (IC_25_) in order to assess its antimigratory potential rather than its cytotoxic effect. After the incubation time, the culture medium from the side channels was removed, and the plant extract, diluted in medium without supplementation, was added to the channel containing the cells. In the opposite channel, culture medium with 20% fetal bovine serum was injected, acting as a chemoattractant. The device was incubated for 24 h.

After this period, cells were stained with Calcein-AM, and images were captured using a fluorescence confocal microscope (Zeiss LSM 700) with 10x magnification. The distance migrated by the cells was analyzed in ImageJ^®^ software. For analysis, the distance traveled by the cell with the highest migratory capacity towards the chemoattractant was measured in seven distinct regions. Results were expressed as mean ± standard error.

### Statistical Analysis

Experiments were conducted in triplicate, and data are presented as mean ± standard error (SEM) from at least three independent experiments. Data normality was assessed using the Shapiro-Wilk test, and the significance of results was determined using the Student’s t-test or analysis of variance (ANOVA), followed by the Dunnett or Tukey post-hoc test. The significance level adopted for all experiments was *p* ≤ 0.05. Data analysis and graph construction were performed using ImageJ and GraphPad Prism version 8.0.

## Results

### Extract of *Ageratum fastigiatum* Reduces the Viability of MDA-MB-231 Cells in 2D Culture Model

To ensure that the cytotoxic effect of the extract was not influenced by DMSO, the solvent used for dilution, a DMSO concentration of 0.250% was established as the negative control. Additionally, doxorubicin was used as a positive control for cell death at its previously determined IC_50_ concentration of 7.32 µg/mL (see supplementary material).

**Fig. 2:**
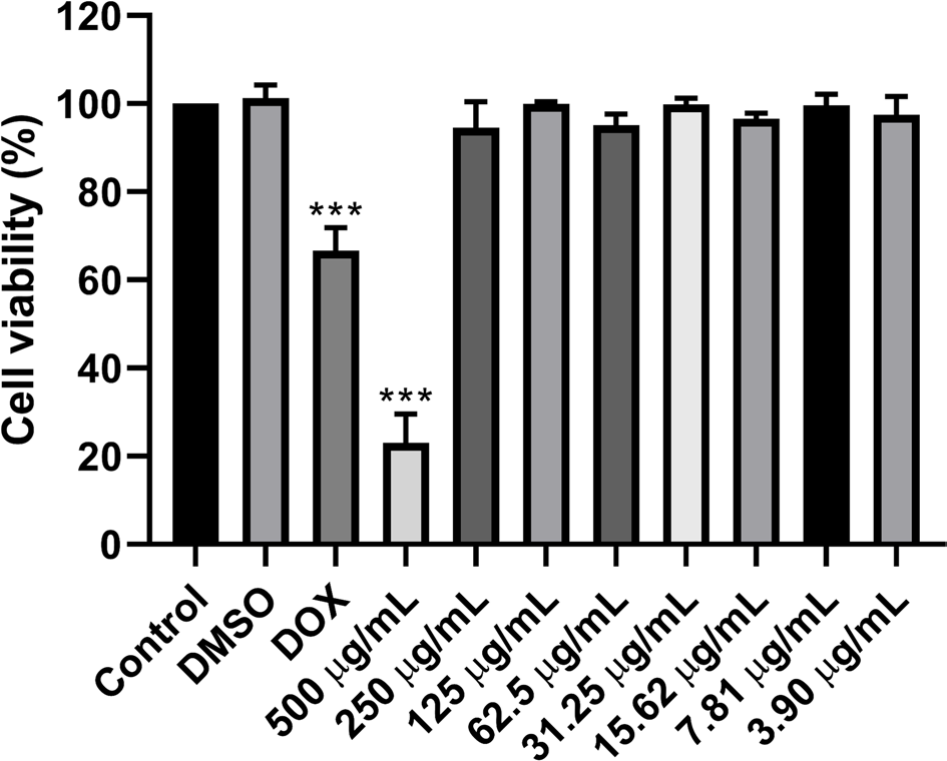
Effect of the ethanol extract of *Ageratum fastigiatum* on the viability of MDA-MB-231 cells quantified by the MTT colorimetric assay. Serial concentrations of the extract, ranging from 500 to 3.90 µg/mL, were tested on both cell lines with a 72-hour exposure to the treatment. The results were compared to the untreated control group and expressed as the mean cell viability ± SEM based on three independent experiments performed in triplicate. Statistically significant differences were determined by ANOVA, followed by the Dunnett post-hoc test, with significance values of ***p < 0.0001. DOX: Doxorubicin

The results indicated that high concentrations of the extract were required to reduce the viability of MDA-MB-231 cells, as shown in Fig. 2. A concentration of 500 µg/mL eliminated approximately 80% of the cancer cells. The anticancer drug used as a positive control showed a cell viability of 66%, both evaluated based on the previously calculated IC_50_ concentration. The solvent control, dimethyl sulfoxide (DMSO), did not significantly alter cell viability.

The IC_50_ value for the plant extract was determined to be 308 µg/mL for the MDA-MB-231 breast cancer cell line. Additional analyses were conducted on MDA-MB-231 cells, including the evaluation of the extract’s inhibitory effect on cell migration and its ability to prevent colony formation.

### Extract of *Ageratum fastigiatum* Inhibits Migration Capacity of MDA-MB-231 Cells in 2D Culture Models

**Fig. 3:**
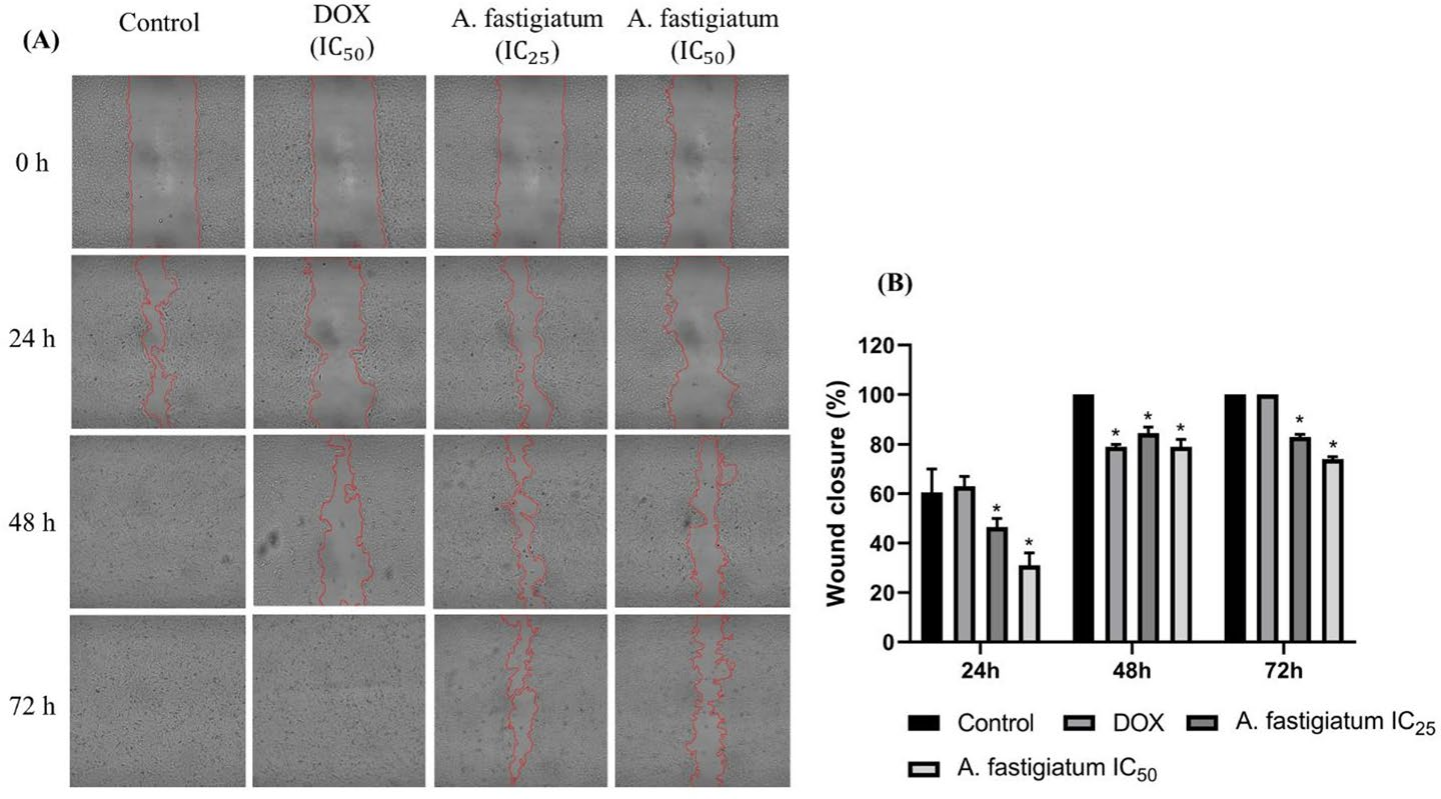
Inhibitory effect of *Ageratum fastigiatum* extract and doxorubicin in MDA-MB-231 cell migration. (**A**) Cells were seeded in 6-well plates, and once a confluent monolayer was reached, a scratch was made using a P-200 pipette tip. Then, the cells were incubated for 72 h with the indicated treatments. The control group was left untreated, while the positive control was treated with doxorubicin (IC_50_). The inhibitory effect of the extract was evaluated at concentrations corresponding to IC_25_ (289 µg/mL) and IC_50_ (308 µg/mL). Images were taken at a magnification of 10x. (**B**) Bar graphs representing the percentage of migration of MDA-MB-231 cells at 24, 48, and 72 h of treatment exposure. The experiment was performed in duplicate (*n* = 2), and the data were expressed as a percentage of wound closure for each condition, representing the mean of two independent experiments ± SEM. Statistically significant differences were determined by ANOVA, followed by Tukey’s post hoc test, with significance values of *p < 0.05. DOX: Doxorubicin

The results showed that treatment with the extract of the plant, at concentrations corresponding to IC_25_ and IC_50_, reduced the migration capacity of MDA-MB-231 cells (Fig. 3A). As illustrated in Fig. 3B, the IC_25_ (289 µg/mL) concentration reduced cell migration by approximately 20% after 48 and 72 h of incubation. The IC_50_ (308 µg/mL) concentration showed a slightly more pronounced effect, with 21% and 26% reductions in scratch area at the same time points, respectively. In contrast, doxorubicin showed a significant initial reduction in the scratch area, with 37% and 21% reductions after 24 and 48 h of treatment, respectively. However, this effect was not maintained over time, and after 72 h of treatment, the cells migrated and closed the wound similarly to the control group.

### *Ageratum fastigiatum* Extract Reduces the Clonogenic Ability of MDA-MB-231 Cells in 2D Culture Model

**Fig. 4:**
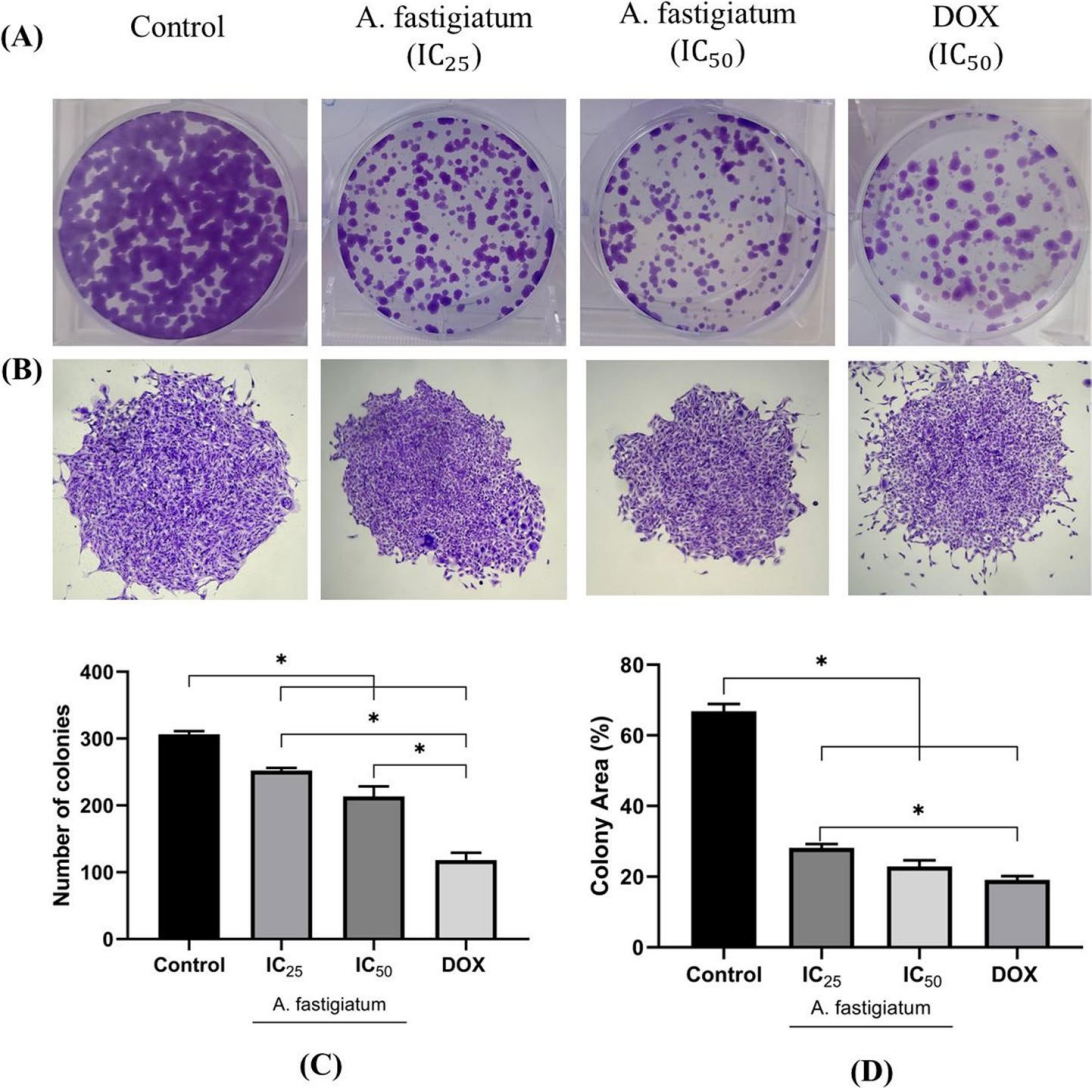
Effect of *Ageratum fastigiatum* extract on the colony formation of MDA-MB-231 cells. (**A**) A density of 500 cells per well were seeded in 6-well plates and incubated for 24 h. Then, the cells were treated with *A. fastigiatum* at concentrations of 289 µg/mL (IC_25_), 308 µg/mL (IC_50_), and doxorubicin (IC_50_) for 72 h. After treatment, the medium was replaced, and the plate was incubated for an additional 12 days and monitored daily. After this period, the colonies were fixed with 3.7% paraformaldehyde and stained with 0.5% crystal violet. The experiment was performed in triplicate (*n* = 3). (**B**) Crystal violet-stained colonies under a 4x magnification. (**C**) Graphical representation of the estimated number of colonies counted with the help of an inverted microscope. (**D**) Bar graph representing the percentage of area formed by colonies in each treatment. Statistically significant differences were determined by ANOVA, followed by the post hoc Tukey test, with significance values of *p < 0.05. DOX: Doxorubicin

The results demonstrated that the treatments investigated visibly reduced colony formation compared to the control group (Fig. 4A). The number of colonies formed was quantified using an inverted microscope. Although no statistically significant differences were observed between the concentrations of the plant extract, the number of colonies formed was approximately 252 colonies for IC_25_ and 213 colonies for IC_50_ (*n* = 3) (Fig. 4C). Doxorubicin treatment showed greater efficacy, significantly reducing the number of colonies to 118 (Fig. 4C). In the control group, around 306 colonies were estimated (Fig. 4C).

Regarding the number of colonies counted, the statistical data revealed a significant difference between the control group and all treatments (*p* < 0.05), indicating the effectiveness of the extract and doxorubicin in inhibiting the clonogenic ability of the cells. Furthermore, a significant difference (*p* < 0.05) was identified when comparing IC_25_ and IC_50_ of the extract with the IC_50_ of doxorubicin, with the standard drug showing a stronger inhibitory effect. However, no statistically significant difference was observed between the IC_25_ and IC_50_ concentrations of the plant extract.

The analysis of the colony area percentage, shown in Fig. 4B and D, reinforced the previous findings, highlighting that both the IC_25_ and IC_50_ concentrations of the *A. fastigiatum* extract, as well as doxorubicin treatment, resulted in a significant reduction in colony area compared to the control group. The data revealed that there was no statistically significant difference (*p* < 0.05) between the cells treated with doxorubicin and those treated with the IC_50_ concentration of the extract. This lack of difference may be explained by the fact that although doxorubicin forms fewer colonies, the colonies formed tend to be larger compared to those formed by the IC_50_ treatment.

On the other hand, the comparison between doxorubicin and the IC_25_ concentration of the extract showed a statistically significant difference, highlighting the superiority of the doxorubicin in reducing colony area. Between the two concentrations of the extract investigated, no significant difference was observed in the colony area, likely due to the antiproliferative effect of the extract may have reached a plateau within the evaluated concentrations.

### *Ageratum fastigiatum* Extract Induces Cell Cycle Arrest in MDA-MB-231 Cells At the G0/G1 Phase

**Fig. 5:**
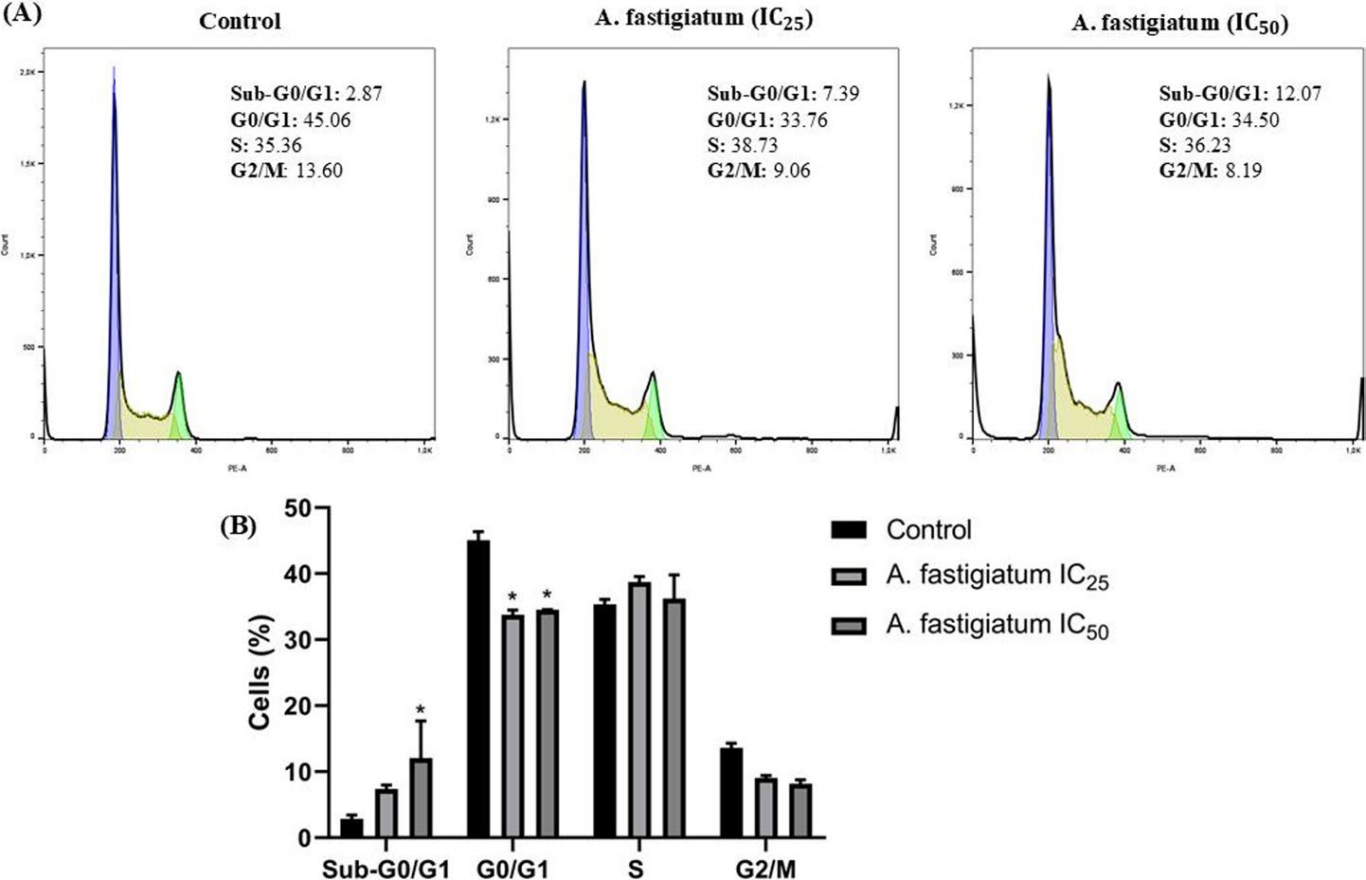
Effect of *Ageratum fastigiatum* extract on the cell cycle of the triple-negative breast cancer cell line MDA-MB-231 after treatment with IC_25_ (289 µg/mL) and IC_50_ (308 µg/mL). Cells were incubated with the plant extract for 72 h, and cell cycle analysis was performed by flow cytometry. Experiments were conducted in triplicate, and data are presented as mean ± SEM. Statistical significance was evaluated using two-way ANOVA followed by Tukey’s post-hoc test (∗*p* < 0.05)

To investigate the mechanism by which *A. fastigiatum* extract inhibited tumor cell proliferation and migration, cell cycle analysis was performed by flow cytometry. The results indicated that the extract induced cell cycle arrest and apoptosis in MDA-MB-231 cells. As shown in Fig. 5, treatment with the IC_25_ concentration increased the sub-G0/G1 cell population to 7.39%, compared to 2.87% in the control, although this difference was not statistically significant. In contrast, the IC_50_ concentration induced a significant increase in the sub-G0/G1 population, reaching 12.07% relative to the control. Additionally, a statistically significant decrease in the G0/G1 cell population was observed following treatment with IC_25_ and IC_50_, corresponding to 33.76% and 34.50%, respectively.

### Cytotoxic Response To Drugs is Different in 3D Culture Models

We assessed the effect of the plant *Ageratum fastigiatum* and doxorubicin on the MDA-MB-231 cell line in a 3D model using a microfluidic device. To mimic the extracellular matrix, a collagen hydrogel mixture (2.5 mg/mL, pH 7.4) was used, with a cell density of 1 × 10^6^ cells/mL. The plant extract was tested at the highest concentration previously evaluated in the 2D model (500 µg/mL; approximately IC_80_) while doxorubicin was tested at its IC_50_ (7.32 µg/mL). The cells were exposed to treatments for 72 h.

**Fig. 6:**
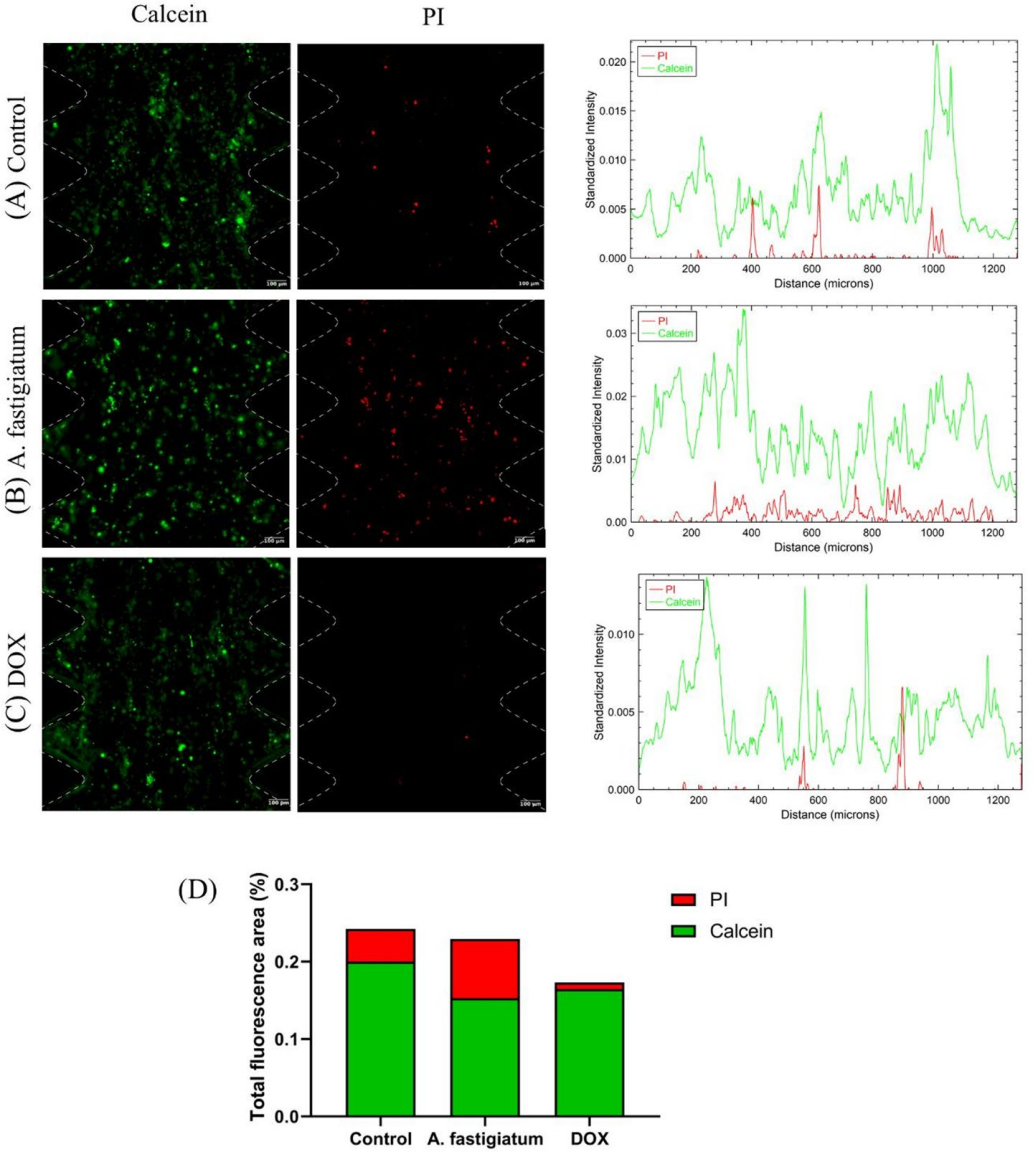
Analysis of MDA-MB-231 cell viability cultured in microfluidic devices. (**A**-**C**) Cancer cells, at a density of 1 million cells/mL, were incorporated into the central microchamber of the device for 3 days to stabilize. After this period, the cells were treated with *Ageratum fastigiatum* extract (500 µg/mL) and doxorubicin (7.32 µg/mL) for 72 h. Cell viability was quantified using Calcein-AM and Propidium Iodide (PI). The data were presented through fluorescence profile graphs for each treatment. The images were captured at 10x magnification. Scale bar: 100 μm. (**D**) The volume occupied by viable and dead cells was estimated, with the results expressed in bar graphs. Viable cells are stained green, while dead cells are stained red. DOX: Doxorubicin and PI: Propidium Iodide

Three days post-treatment, the tumor cells were stained using a live/dead viability kit. Green staining, resulting from calcein-AM labeling, indicates the presence of live cells, while red staining, positive for propidium iodide (PI), marks dead cells due to loss of membrane integrity. The results, expressed in fluorescence intensity profiles (Figs. 6A-C), demonstrated that the extracellular matrix played a significant role in the response to the treatments. Fluorescence profiling figures revealed that both the plant extract and doxorubicin did not exhibit high cytotoxic effects compared to the results observed in 2D cultures from the MTT assays.

The area occupied by the live and dead cells, estimated from slices of a region of interest, is represented by bar graphs (Fig. 6D). The results correspond to the sum of the total area occupied by the cells in the region occupied by 10 slices. The data were presented as the percentage of the area occupied by each marker relative to the total image area, resulting in a percentage of the area occupied by the cells. The relation between the results of the total area occupied by cells, along with the fluorescence intensity profiles, showed that the treatments evaluated did not exhibit effects similar to those observed in the 2D culture models for MDA-MB-231 cells. For viable cells, the percentage of total area of fluorescence was approximately 20%, 15%, and 16% for the control, plant treatment, and doxorubicin, respectively. For dead cells, the values were 4%, 8%, and 1%, respectively.

### *Ageratum fastigiatum* Extract Reduces Migration of MDA-MB-231 Cells in 3D Culture Models

**Fig. 7:**
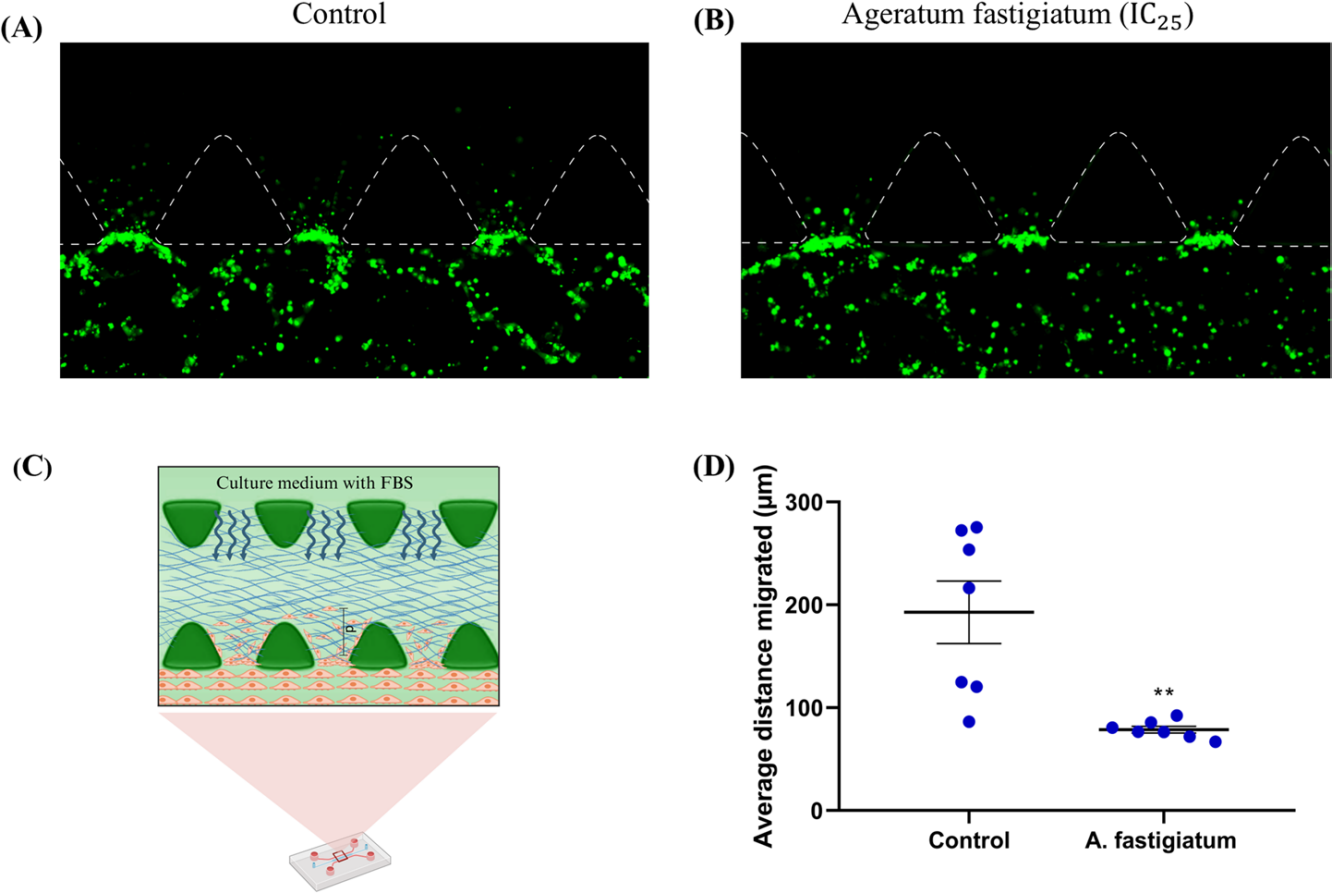
Migratory capacity of MDA-MB-231 cells in 3D culture models. (**A** and **B**) Fluorescence images illustrating the distance migrated by cancer cells in the control and *A. fastigiatum* ethanol extract-treated groups. The images were taken at 10x magnification. (**C**) Representative image of the measurement of the migrated distance for each cell. d: Distance. Created in https://BioRender.com. (**D**) Results of the average distance migrated by MDA-MB-231 cells. Statistically significant differences were assessed using the Student’s t-test, with significance values of **p < 0.001

A study was conducted to assess the inhibitory effect of the ethanol extract of *Ageratum fastigiatum* on the migration of MDA-MB-231 cells in a 3D culture model. The cancer cells were inserted into one of the lateral channels of the microfluidic device, and their ability to invade the collagen hydrogel (2.5 mg/mL) in the central microchamber was analyzed 24 h after treatments, as illustrated in Fig. 7A and B. The average migrated distance was quantified in seven distinct regions, and the data are presented in Fig. 7C and D as mean ± SEM.

The tested concentration of the extract was 289 µg/mL, equivalent to the IC_25_. The IC_50_ was not evaluated since the results obtained in the scratch assay were similar to the IC_25_. The results indicated that the MDA-MB-231 cells in the control group migrated an average distance of 192.73 ± 28.07 μm, while the cells treated with the plant extract migrated an average distance of 78.61 ± 2.97 μm (t-test, *p* < 0.05). This suggests that the plant extract inhibited the migratory capacity of breast cancer cells by approximately 59.23% compared to the control group, resulting in a mean difference in distance of 114.1 ± 30.48 μm.

## Discussion

Triple-negative breast cancer (TNBC) is one of the most aggressive and lethal subtypes of breast cancer, characterized by the absence of estrogen, progesterone, and HER2 receptors, which limits treatment options to conventional chemotherapy [17]. Its high metastatic potential is associated with the production of proteolytic enzymes, such as matrix metalloproteinases, that degrade components of the extracellular matrix and facilitate tissue invasion [18]. In this context, medicinal plants have emerged as promising alternatives due to their bioactive properties, including antitumor, anti-inflammatory, and antioxidant activities, which can interfere with key mechanisms of tumor progression such as cell proliferation, angiogenesis, migration, and metastasis [19–24]. In the Brazilian Cerrado, especially in the southeastern region of Minas Gerais, the plant *Ageratum fastigiatum* (Gardn.) R.M. King & H. Rob., stands out for its therapeutic potential, although still little explored [25]. While previous studies have reported its antioxidant, anti-inflammatory, and antimicrobial activities [26], its antitumor effects have not yet been investigated, highlighting the importance of the present study.

This study aimed to evaluate the antitumor effect of the ethanolic extract from the aerial parts of *Ageratum fastigiatum* in triple-negative breast cancer cells. Additionally, the ability of the extract to inhibit cell migration and reduce colony formation was investigated, as these are crucial factors in tumor progression.

The results of 2D cultures showed that treatment with the extract of *A. fastigiatum* exhibited a cytotoxic effect at high concentrations in triple-negative breast cancer cells, suggesting a dose-dependent response to toxicity. Cell viability at the highest concentration tested was approximately 23%.

As a preliminary evaluation of the potential of microfluidoc devices for in vitro drug testing and the effect of the extracellular matrix in 3D culture models on drug response, we used the highest tested concentration for *A. fastigiatum* extract and doxorubicin (at IC_50_) in MDA-MB-231 cells. The fluorescence profile and total cell area analysis showed that the plant extract (at IC_80_) had a greater cytotoxic effect than doxorubicin (at IC_50_), as evidenced by a lower live cell area and a higher percentage of cell death. As expected, the total fluorescence area of live cells in the control group was higher than that of dead cells and greater than in the treated groups. Additionally, the total area of both live and dead cells in the plant extract and doxorubicin groups was lower than in the control, suggesting an antiproliferative effect. This agrees with previous clonogenic assay results, where both treatments suppressed colony formation. The difference in drug responses between 2D and 3D models could be attributed to the presence of collagen hydrogel, which mimics the human extracellular matrix and acts as a physical barrier, limiting homogeneous drug diffusion and cell contact within the device.

Doxorubicin’s IC_50_ did not show similar cytotoxicity in 3D cultures compared to 2D models. Differences in drug effects between 2D and 3D models were also observed by other researchers. A study using ovarian cancer spheroids showed significantly greater resistance to doxorubicin and topotecan after 72 h compared to 2D cultures, with spheroids being 3.09 times more resistant [27]. In a separate study, doxorubicin effectively reached the central region of colon cancer cells (HCT-116) in microfluidic devices with collagen hydrogel after 24 h [28]. The chemical composition of plants is essential for conferring their antitumor properties, highlighting the importance of identifying the most abundant bioactive components in each species.

The phytochemical analysis of the plant used in this study revealed the presence of 11 monoterpenes and 15 sesquiterpenes, with emphasis on α-pinene, limonene, trans-caryophyllene, α-humulene, caryophyllene oxide, humulene 1,2-epoxide, 1,6-humuladien-3-ol, and α-cadinol [29, 30]. The leaves used for the preparation of the extract in this study were obtained from the same collection used by researcher Lucas [31], in which the phytochemical analysis of the ethanolic extract fraction identified two flavonoids previously undescribed in the plant’s chemical composition, 7-methylquercetin and rhamnocitrin. The presence of 7-methylquercetin could contribute to the observed cytotoxic effects of the extract [31]. In previous studies, these compounds have been shown to reduce cell proliferation and inhibit tumor cell migration and invasion [32, 33].

Triple-negative breast cancer cells have the ability to invade neighboring tissues and spread to other organs, promoting metastasis. This process involves the migration of cells to adjacent tissues, the formation of new tumors, and, consequently, an increased risk of mortality in patients with cancer [34, 35]. In this context, recent studies have used the scratch assay to evaluate the inhibitory potential of natural products on the migration of cancer cells due to its simplicity and the reliability of the results.

Based on this, we used the scratch assay to investigate the ability of the extract to reduce the migration of MDA-MB-231 cells. In the experiment, we evaluated the antimigratory effect of doxorubicin at a concentration of IC_50_ and the plant extract at concentrations of IC_25_ and IC_50_. The results showed that the extract, at both tested concentrations, reduced cell migration by approximately 20% and 26% at 48 h, and the inhibitory effect remained similar up to 72 h. This finding is significant because it could indicate the potential of the plant to reduce tumor metastasis, one of the major factors associated with the high mortality rate of triple-negative breast cancer.

Additionally, a study on the migration of cancer cells was performed in 3D culture models after 24 h of treatment with *A. fastigiatum* (IC_25_). The results showed that the plant extract significantly inhibited the migration of tumor cells compared to the control group, corroborating the findings of the scratch assay. Similar studies that investigated the migration of MDA-MB-231 cells in 24 h obtained results consistent with our study [36]. The authors observed cancer cells, under similar experimental conditions and the same period of time. We concluded that triple-negative breast cancer cells have high migratory capacity, and that *A. fastigiatum* extract was effective in significantly reducing this migration. Diminished cell migration can be explained at least partially by the previously observed reduction of the expression of CD18 caused by *A. fastigiatum* [37]. CD18 is a membrane adhesion integrin belonging to the $\:{\beta\:}_{2}$-integrin subfamily important for migration.

In the scratch assay, doxorubicin inhibited the migration of MDA-MB-231 cells by 63% and 79% at 24 and 48 h, respectively. After 72 h, the cells migrated and closed the wound. Interestingly, some studies have shown that doxorubicin can increase the migratory and invasive capacity of breast cancer cells by activating IKKs and Myc, which may explain the wound closure after 72 h [38, 39]. This highlights the importance of investigating the combination of *Ageratum fastigiatum* extract with doxorubicin. Combining *A. fastigiatum* extract with doxorubicin could offer a promising approach to reduce cancer cell migration and enhance the cytotoxic effects of both treatments.

The clonogenic assay revealed that both concentrations of *A. fastigiatum* extract tested promoted a reduction in the number of colonies formed by approximately 18.0% and 31.0%, respectively, compared to the untreated group, indicating the extract ability to inhibit cell proliferation and the formation of colonies from a single cell. Additionally, both concentrations of the extract showed similar effects, both in the number of colonies counted and in the percentage of the area occupied by the colonies, indicating that the extract exerts an antiproliferative effect, being more effective at higher concentrations, as also observed in the MTT assay. The lack of a significant difference between IC_25_ and IC_50_ suggests that the effect of the extract at these concentrations may be reaching a plateau, indicating a possible saturation in the mechanism of action.

Doxorubicin had a significant inhibitory effect on the number of colonies formed. However, despite the drastically reducing the number of colonies observed, the colonies formed exhibited a considerable percentage of area, meaning that large colonies were formed, which did not differ significantly from the area observed at the IC_50_ concentration of *A. fastigiatum* extract. Although doxorubicin formed fewer colonies compared to the extract, the colonies formed exhibited a larger area than those formed under the extract treatment. This result suggests a complementary potential between the two treatments. While doxorubicin was effective in reducing the total number of colonies formed, *A. fastigiatum* extract not only contributed to the reduction in the number of colonies but could also help limit the size of the colonies. Therefore, the combination of both treatments could result in a synergistic effect, combining the potent antiproliferative action of doxorubicin with the ability of *A. fastigiatum* extract to limit colony growth. Future studies to evaluate this therapeutic interaction in more complex experimental models, such as co-culture or in vivo models, would be crucial to validate this hypothesis and explore the clinical potential of this combination.

The antiproliferative effect of *Ageratum fastigiatum* extract on MDA-MB-231 cells can be explained, at least in part, by its ability to induce cell cycle arrest at the sub-G0/G1 phase. In our study, a significant increase in the sub-G0/G1 population was observed following treatment with the extract, suggesting nuclear fragmentation followed by apoptosis. This finding is consistent with previous reports demonstrating that natural products can promote DNA fragmentation, leading to the accumulation of cells in the sub-G0/G1 phase, which is considered a classical marker of apoptosis [2, 40, 41]. Furthermore, we observed a reduction in the G0/G1 cell population, indicating that cells which would normally progress through this phase were redirected to the sub-G0/G1 stage. Taken together, these results suggest that the cytotoxic activity of *A. fastigiatum* extract involves the induction of apoptotic pathways and disruption of normal cell cycle progression, thereby contributing to the inhibition of tumor cell proliferation.

This study provides relevant insights into the antimigratory and cytotoxic effects of *A. fastigiatum* extract in tumor models. Considering the complexity of the biological processes involved, in-depth experimental investigations of the underlying molecular mechanisms, such as the analysis of specific apoptotic and migratory pathways, were not conducted within the scope of this work. In addition, the study presents experimental limitations, including the lack of assessment of concentration gradients in three-dimensional models, the absence of phytochemical characterization and quantification of bioactive compounds, and the lack of testing in non-tumorigenic cell models, necessary to evaluate selectivity, safety, and therapeutic potential. Future investigations are therefore needed to completely understand the effects and therapeutic potential of *A. fastigiatum* extract.

## Conclusion

This study demonstrated that the ethanolic extract of *Ageratum fastigiatum* exerts cytotoxic, antiproliferative, and antimigratory effects on triple-negative breast cancer cells (MDA-MB-231) in vitro models. In two-dimensional cultures, the extract reduced cell viability at higher concentrations, inhibited cell migration, decreased colony formation, and induced cell cycle alterations consistent with apoptotic processes, indicating interference with cellular mechanisms associated with tumor growth and progression. Evaluation of the extract in a three-dimensional microfluidic tumor-on-a-chip model revealed responses that differed from those observed in conventional 2D systems, highlighting the influence of the tumor microenvironment on treatment efficacy. The presence of a collagen-based extracellular matrix emerged as a key factor modulating cellular responses by affecting compound diffusion and cell–matrix interactions. Collectively, these findings support the potential of *A. fastigiatum* as a source of bioactive compounds with antitumor activity and emphasize the importance of incorporating advanced three-dimensional models for a more physiologically relevant assessment of therapeutic candidates. Nonetheless, it is important to implement other molecular and immunohistochemical analyses to investigate the mechanisms of action of the plant extract to reduce tumor cell viability, migration and proliferation.

## Supplementary Information

Below is the link to the electronic supplementary material.

Supplementary Material 1 (DOCX 126 KB)

## Data Availability

The data that support the findings of this study are available on request from the corresponding author.
