# Supplementary material for: Effect of Ageratum fastigiatum on Viability, Migration and Proliferation of Breast Cancer Cells in 2D and 3D Culture Models
Source: Appl Biochem Biotechnol. 2026 Feb 7;198(4):3061–80. doi: 10.1007/s12010-026-05589-x (PMC13032935; doi:10.1007/s12010-026-05589-x)
Supplement: Supplementary file 1 — Supplementary Material 1 (DOCX 126 KB) [file 12010_2026_5589_MOESM1_ESM.docx]

**
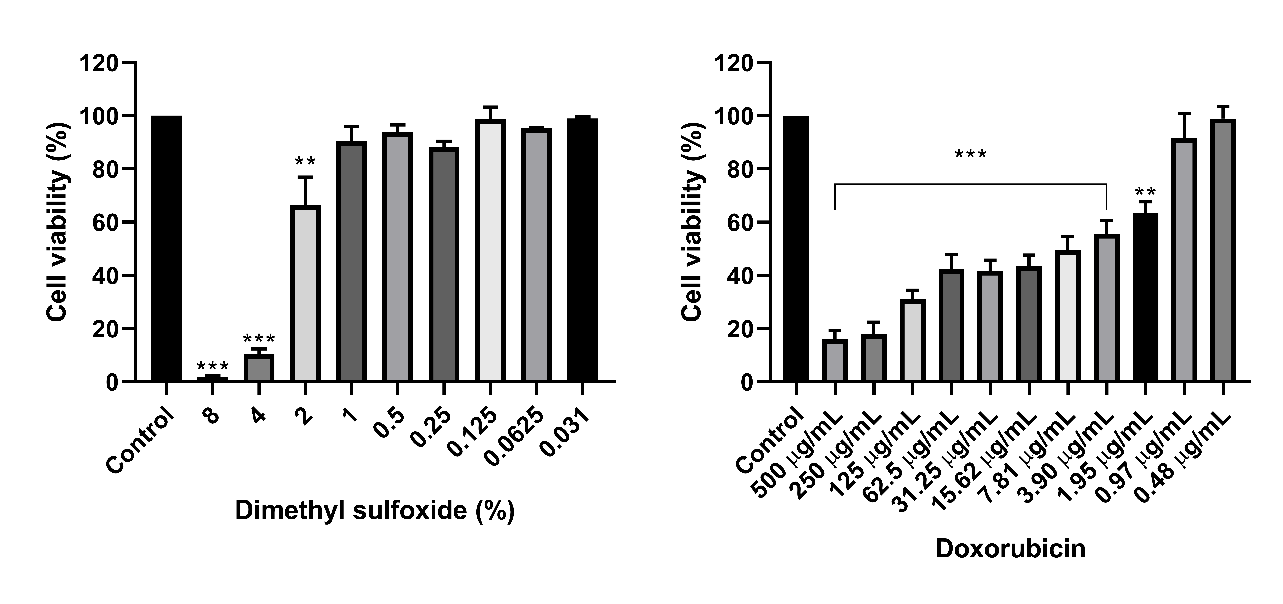
**

**(B)**

**(A)**

Percentage of cell viability of MDA-MB-231 cells after 72 hours of incubation with (A) dimethyl sulfoxide (DMSO) at concentrations ranging from 0.031% to 8% and (B) doxorubicin (DOX) at concentrations ranging from 0.48 to 500 μg/mL. Cell viability was assessed using the MTT colorimetric assay. Data are presented as the mean ± SEM from three independent experiments, each performed in triplicate. Statistical significance was evaluated by one-way ANOVA followed by Dunnett’s post hoc test (**p < 0.001; ***p < 0.0001).
